# Development of the Heterodyne Laser Encoder System for the X–Y Positioning Stage

**DOI:** 10.3390/s21175775

**Published:** 2021-08-27

**Authors:** Chung-Ping Chang, Tsung-Chun Tu, Siang-Ruei Huang, Yung-Cheng Wang, Syuan-Cheng Chang

**Affiliations:** 1 Department of Mechanical and Energy Engineering, National Chiayi University, Chiayi 600, Taiwan; s1080384@mail.ncyu.edu.tw (T.-C.T.); s1090381@mail.ncyu.edu.tw (S.-R.H.); 2 Department of Mechanical Engineering, National Yunlin University of Science and Technology, Yunlin 640, Taiwan; wangyc@yuntech.edu.tw (Y.-C.W.); d10911001@yuntech.edu.tw (S.-C.C.)

**Keywords:** laser encoder, heterodyne laser interferometer, geometric error, positioning stage

## Abstract

This investigation develops a laser encoder system based on a heterodyne laser interferometer. For eliminating geometric errors, the optical structure of the proposed encoder system was carried out with the internal zero-point method. The designed structure can eliminate the geometric errors, including positioning error, straightness error, squareness error, and Abbe error of the positioning stage. The signal processing system is composed of commercial integrated circuits (ICs). The signal type of the proposed encoding system is a differential signal that is compatible with most motion control systems. The proposed encoder system is embedded in a two-dimensional positioning stage. By the experimental results of the positioning test in the measuring range of 27 mm × 27 mm, with a resolution of 15.8 nm, the maximum values of the positioning error and standard deviation are 12.64 nm and 126.4 nm, respectively, in the positioning experiments. The result shows that the proposed encoder system can fit the positioning requirements of the optoelectronic and semiconductor industries.

## 1. Introduction

Nowadays, the semiconductor and optoelectronics industries are some of the most important industries worldwide. The precision two-dimensional positioning system is the key to the next generation in those industries. For example, mask aligner, die bonder [1,2,3,4], and laser cutting technology. The features of the positioning stage are highly related to the encoding system. A high-accuracy positioning stage needs an encoding system with the resolution of the sub-micrometer to the nanometer scale, and the assembly conditions of the encoding system should be restricted strictly.

One of the main components of a precision positioning system is a high-end encoder, such as a laser encoder based on interferometer technology. Since the 1960s, interferometer technology has been utilized in the precision length measurement of a large range, when T. H. Maiman invented the first commercial laser tube [5]. In 1983, Conférence Générale des Poids et Mesures (CGPM) defined 1 m as “the distance traveled by light in a vacuum in 1/299792458 s”. Since then, the wavelength of light has become the basic definition of length [6]. Therefore, various novel laser interferometers and laser encoders are proposed constantly [7,8]. Moreover, they are widely used in calibration and positioning tasks.

Laser encoders can be sorted into homodyne interferometers [8,9] and heterodyne interferometers [10,11], according to the laser source. Both of these interferometers have good performance in the field of precision positioning. In general, the heterodyne interferometer system has a better signal-to-noise ratio (SNR). Therefore, the heterodyne system has a robust signal; this is one of the most significant characteristics for an interferometer system to operate in ordinary conditions. The displacement information of the heterodyne interferometer is attached to the frequency shift signal. This reason that the heterodyne signal is insensitive to the amplitude of the laser intensity. Therefore, the heterodyne interferometer is more robust compared with the homodyne interferometer. For this reason, this study will use the heterodyne system for its outstanding signal quality. The major signal processing for the heterodyne interferometer is the phase-detection module [12,13,14]. The phase-detection technology, which is included in the lock-in amplifier, has been commercialized by Robert H. Dicke. It is widely used in the signal processing field for the high SNR.

In the research field of precision positioning technology, there are many ways to reduce geometric errors. Some technologies focus on the compensation of single-axis geometric errors [15,16]. These methods are simple, but they cannot compensate for the interaction error of multiple axes. Another part of the technology focuses on multi-axis volumetric error compensation [17,18,19]. This method can solve the above-mentioned interactive errors, but the error analysis algorithm is complicated and cannot implemented in most commercial controllers. In this study, a method based on the Abbe principle has been utilized [20]. In 2003, Theo A.M. Ruijl and Jan van Eijk designed an optical, mechanical structure that can eliminate the geometric errors of the machines [21]. The design of the positioning stage in this reference has three laser interferometers that are aligned in x, y, and z directions. This design is based on the Abbe principle, which can reduce the geometric error for the positioning system; this is one of the milestones of precision positioning technology. Since then, much research on precision positioning and measuring machines is based on this design [22,23]. These error-reduction technologies are achieved through the optical, mechanical design of the stage and laser encoders. The comparisons between the proposed laser encoder system and those existing designs in the relevant references are presented in Table 1.

Based on the above, this research focuses on the two-dimensional positioning issue and proposes a laser encoder system for the precision X–Y stage. The arrangement of the optical, mechanical structure effectively reduces various geometric errors. The novelty of this research is the optical, mechanical design and signal processing. This design can simultaneously meet the requirements of the zero-point method, embedded optical system, heterodyne system, shared reference signal, and differential signaling. In order to realize the precise positioning task, the proposed encoder system is embedded in a two-dimensional positioning stage. Moreover, a series of positioning experiments have been carried out in this research.

## 2. Principle and Theory

In this section, a two-dimensional laser encoder system is introduced. The mechanical and optical designs are integrated into this system to achieve a simplified heterodyne laser encoder system. The signal processing system includes an automatic gain control module, a phase-detection module, and an interpolation module. The details of the fundamental principle and theory are revealed as follows.

### 2.1. Geometric Error-Reducing Method

During the movement of the positioning stage, various geometric errors will occur, due to the assembly conditions of the mechanical components. Many examples of error reduction and compensation are proposed in the previous research [21,22,23]. However, due to the strict requirement of assembly and calibration, it cannot be widely used in the industry. For example, it is not easy to precisely overlap multiple laser beams at one point. For this reason, this manuscript proposes an optical, mechanical design, to improve the positioning accuracy of the stage in a convenient and low-cost way. The error-reduction method of this research is based on the internal zero-point method [24]. Compared with the previous research, the proposed optical, mechanical structure has a simplified design and a robust signal. In this way, this can reduce the geometric errors, including positioning errors, straightness errors, squareness errors, and Abbe errors.

**Figure 1:**
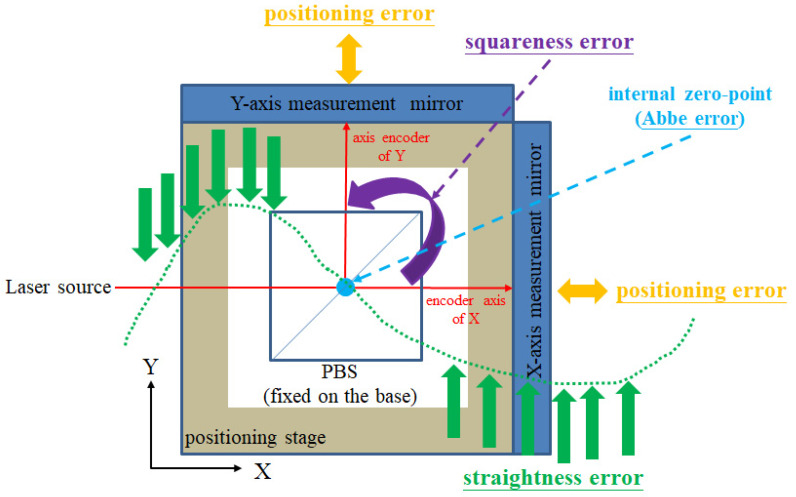
Geometric error-reduction mechanism.

Figure 1 shows the internal zero-point method used in this manuscript. The following are the explanations of the geometric error-reduction mechanism. It is a well-known method [15,16], by which the positioning error of the X or Y axes can be reduced by the two laser encoders, which are represented by the orange arrows. The straightness error is induced by the geometrical error of the linear guide in the stage. Due to the optical, mechanical arrangement, this specific straightness error will be measured by the interferometer that is set up in the opposite axis, and compensated for by the closed-loop control system, which is represented by the green arrow. A misalignment of the linear axes will cause the squareness error of the stage. The squareness error can be eliminated by a fine-tuned polarizing beam splitter (PBS), which is represented by the purple arrow. At last, when an internal zero-point is established and is set as the operating position [21,24], the Abbe error can be minimized, which is represented by the blue point. Therefore, this research proposes a laser encoder system with the error-reduction design and heterodyne signal processing.

### 2.2. Introduction of the Interferometric Technology

Nowadays, interferometry plays an important role in many scientific research fields. We can roughly sort interferometers into the following two types: One is the homodyne interferometer structure. Another is the heterodyne interferometer structure. The technologies of the homodyne and heterodyne interferometers are well known [11,15,25,26]. In the following section, the characteristics of these two interferometer structures will be discussed.

#### 2.2.1. Homodyne Interferometer

**Figure 2:**
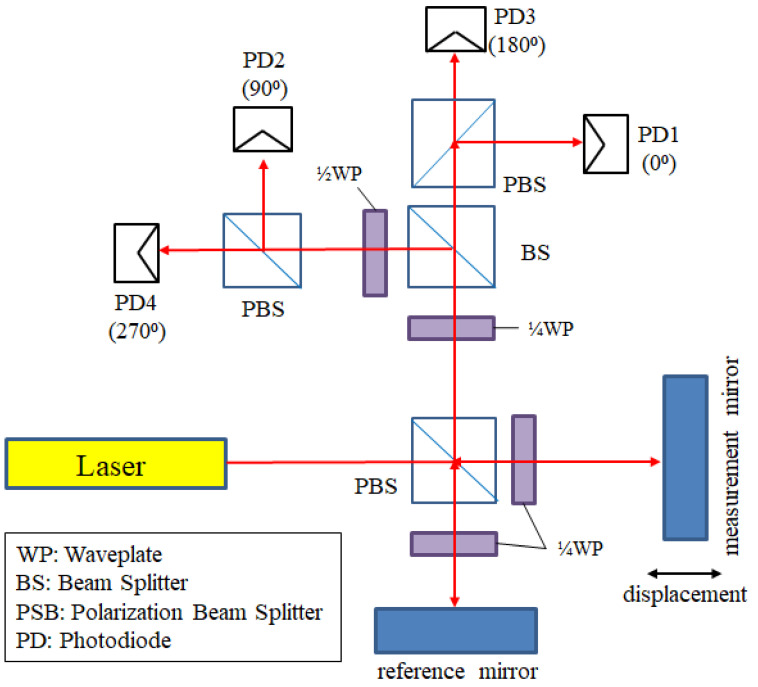
The homodyne interferometer.

The homodyne interferometer operates with a laser source of a single wavelength. A light-splitting mechanism is arranged in the optical structure, to split the laser source into two laser beams. These laser beams are the reference beam and measurement beam, respectively. The splitting mechanism could be the function of amplitude splitting or polarization splitting. After overlapping, these interfering beams are detected by the photodiodes (PDs) at the end of the optical paths. The interference signal that is measured by this detector is a spatial function that can represent the variation in the optical path difference. In this way, the displacement of the object that is attached by the measurement mirror can be measured, as shown in Figure 2. The differential signal processing can reduce the noise for the homodyne interferometers [27], which is introduced in Section 2.2.4. However, the homodyne interferometer needs a complicated optical structure for generating the differential signals. Therefore, this research is based on the heterodyne interferometer, which is introduced in the next section.

#### 2.2.2. Heterodyne Interferometer

**Figure 3:**
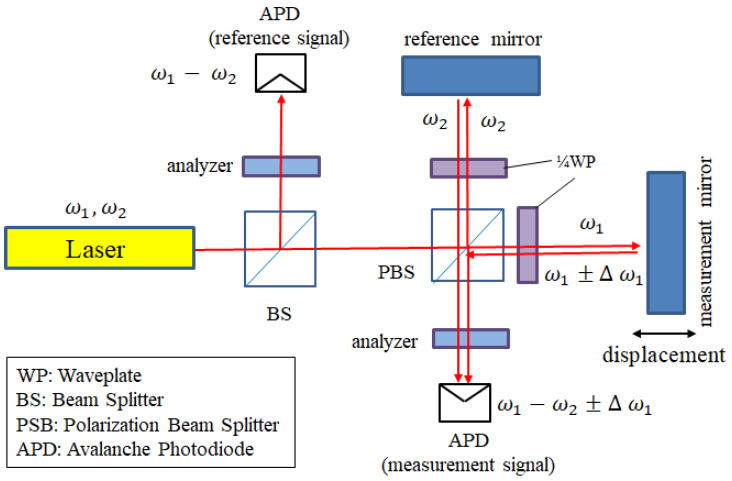
The heterodyne interferometer.

Unlike the homodyne interferometer, the heterodyne interferometer uses a dual-frequency laser source. This laser source has orthogonally polarized beams with different angular frequencies (ω_1_, ω_2_). The basic structure of the heterodyne interferometer is shown in Figure 3 [26]. The movement of the measuring mirror will induce a phase shift (Δω_1_). The displacement information is attached to this phase shift signal. After the phase-detection processing, the displacement can be obtained. The advantage of heterodyne detection technology is that it uses frequency modulation, which can reach a high SNR, and is not susceptible to the influence of the external environment and mechanical errors.

#### 2.2.3. Phase-Detection Technology

**Figure 4:**
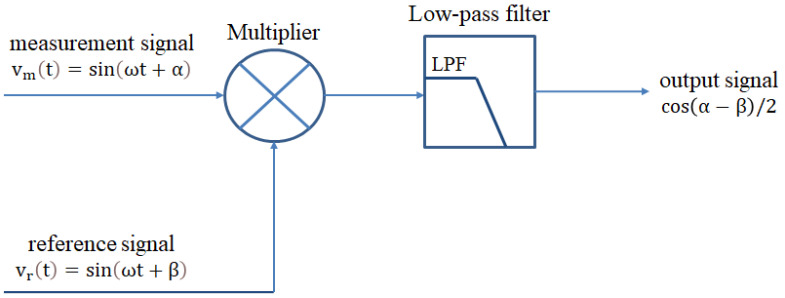
Phase-detection technology.

In this study, the phase-detection technology in the lock-in amplifier will be employed as part of the signal processing, as shown in Figure 4 [12,13,14]. Compared to the commercial lock-in amplifier system, the phase-detection module of the signal processing has no phase-locked function. A multiplier and a low-pass filter will be series-connected as a phase-detection module. The measurement ($v_{m}\left(t\right)$) and the reference ($v_{r}\left(t\right)$) signals are dealt with a multiplier module, and the multiplied result is shown as Equation (1), where ω is the beat frequency, α is the phase of the measurement signal, β is the phase of the reference signal, and t is the time. This result can be divided into two parts. One is the alternating current (AC) term (ωt). Another is the direct current (DC) term, which can indicate the phase difference (α−β). After the signal passing through the low-pass filter, the filter will block the AC term. Therefore, the phase difference can be easily measured by the output signal. This technology is usually used to measure the phase difference in heterodyne laser interferometers.

(1) $$\sin\left(\omega t+\alpha \right)\cdot \sin\left(\omega t+\beta \right)=\frac{1}{2}\left[\cos\left(\alpha -\beta \right)-\cos\left(2\omega t+\alpha +\beta \right)\right]$$

#### 2.2.4. Single-Ended and Differential Signal

A single-ended signal is taken as the voltage difference between a positive wire and the ground. It is the simplest and most commonly used method of transmitting a signal through a wire. The advantage of the single-ended signal is that the impedance is easy to calculate. On the other hand, the noise is only on the positive wire and will be measured along with the output voltage from the sensor. This is an obvious disadvantage when the single-ended devices operate in a factory environment with electromagnetic noise.

**Figure 5:**
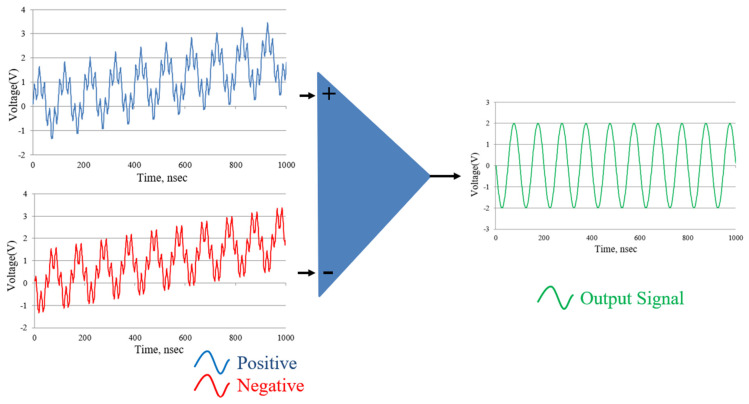
Schematics of the differential signal processing.

On the other hand, a differential signal has no reference to the ground. The measurement is taken as the voltage difference between the positive and negative wires. The advantage of a differential measurement is noise rejection, because of the common-mode rejection of the data acquisition system [28,29], which is shown in Figure 5.

The processing principle of the differential signal is to subtract one signal from the other, which has the same amplitude and opposite phase, which is expressed by Equation (2). When the positive and negative signals are affected by the same external noise, this noise can be rejected by the above method, which can be shown as Equation (3).

(2) $$V_{\mathrm{differential}}=V_{\mathrm{Positive}}-V_{\mathrm{Negative}}$$

(3) $$V_{\mathrm{diff}}=(V_{\mathrm{Positive}}+V_{\mathrm{Noise}})-\left(V_{\mathrm{Negative}}+V_{\mathrm{Noise}}\right)=V_{\mathrm{Positive}}-V_{\mathrm{Negative}}$$

Because the differential signal has the feature of common-mode rejection of the voltage offset (DC term) and the high-frequency noise (AC term), the signal can operate with a high SNR. This differential signal processing is employed in the proposed system, to deal with the signal before it is sent into the driver and controller.

**Figure 6:**
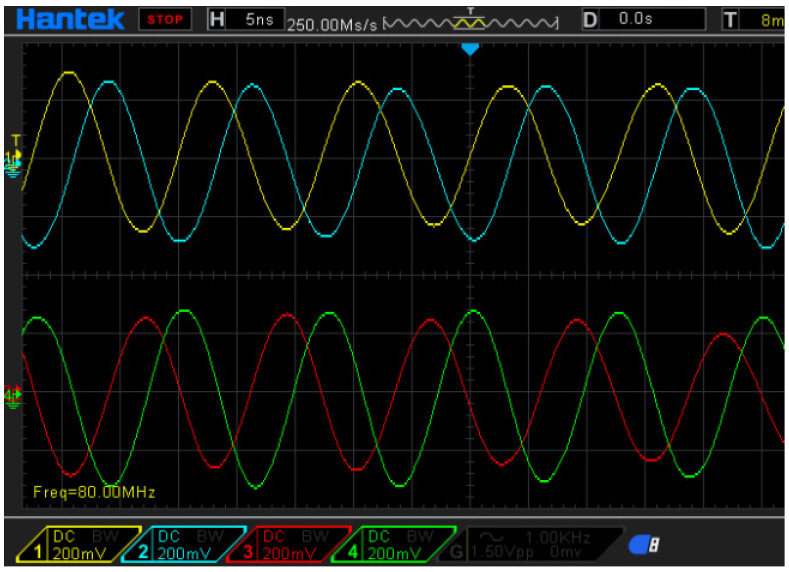
Phase-shifted reference signals of the proposed interferometer.

In this study, the reference signal will be converted into four reference signals, with different phases of 0, 90, 180, and 270 degrees. These shifted phases are chosen to be the same as the signal phase in the commercial encoders. Figure 6 shows the differential reference signals of the proposed laser encoder, with a frequency of 80 MHz. These reference signals are, respectively, sent to the phase-detection module with the measurement signal. After the phase-detection module, the output signals with the different phases can be measured. Therefore, the differential signals, which can reveal the displacement information, can be obtained.

## 3. Design of Proposed Laser Encoder System

In this section, the concept and design of this research have been described. The laser source module, interferometer system, and signal processing module will be discussed separately. Some of the test in the early stage is also revealed as follows.

### 3.1. Heterodyne Laser Source

**Figure 7:**
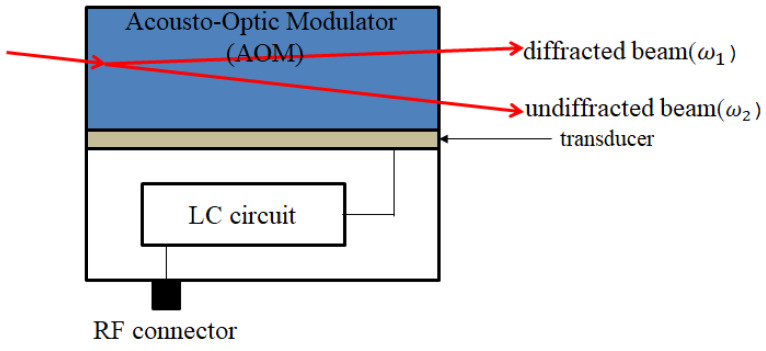
Acousto-optic modulator.

**Figure 8:**
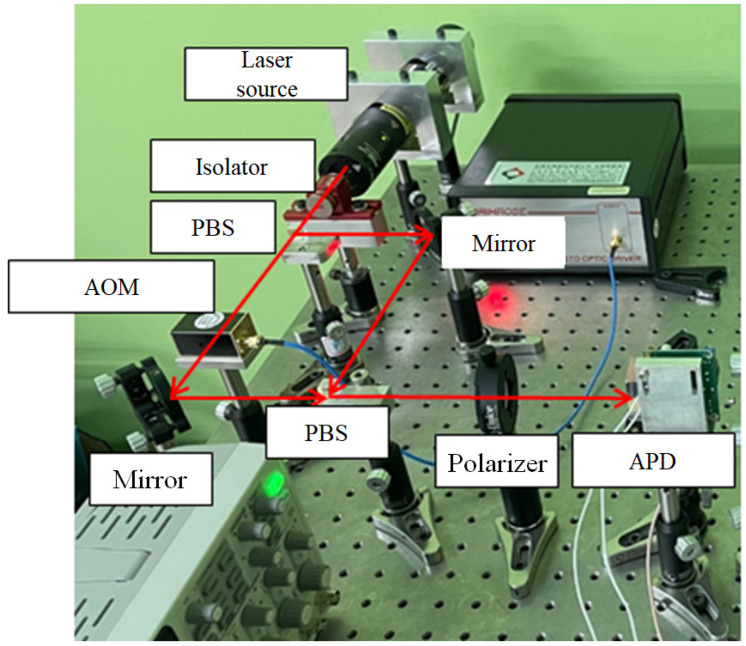
Heterodyne laser source.

**Figure 9:**
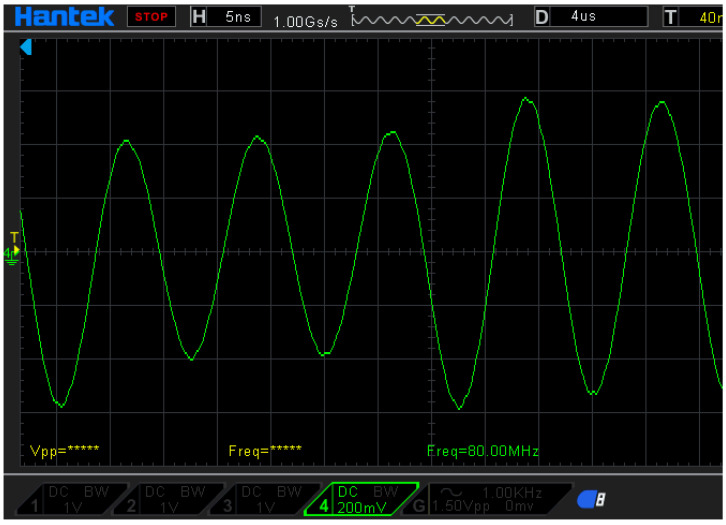
Beat frequency of the heterodyne laser source.

This research uses an acousto-optic modulator (AOM) for the frequency shifting of the laser source, which is shown in Figure 7. The frequency difference between the original and shifted frequency is called the beat frequency. The testing structure of the laser source is shown in Figure 8, and its result is show in Figure 9. The beat frequency of 80 MHz can be measured by the oscilloscope.

### 3.2. Optical Structure

**Figure 10:**
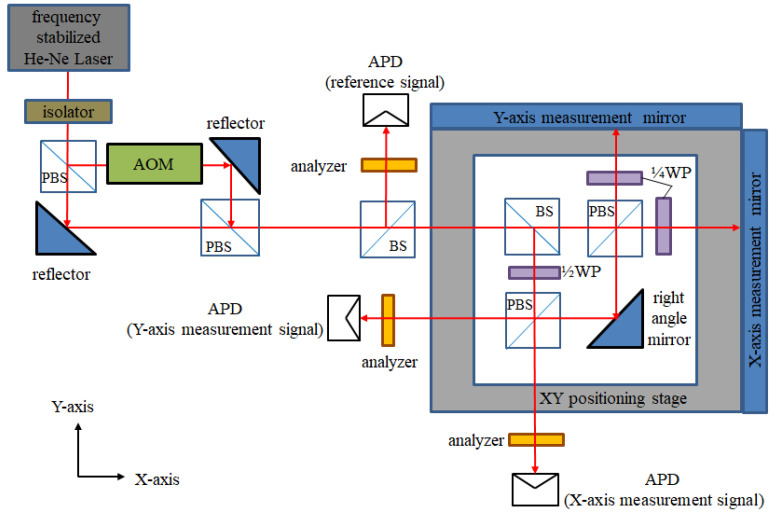
Design of optical structure.

The optical structure of this research is shown in Figure 10. The laser of this system is a frequency-stabilized helium-neon laser, with a wavelength of 633 nm. First, the laser source is divided into two laser beams by a PBS, and one of the laser sources is passed through the AOM. An 80 MHz frequency difference is generated via the AOM. Then, the laser beams transmit to another PBS, and the beams with the original and shifted frequencies are aligned to overlap with each other. In this way, a heterodyne laser source with the modulated beat frequency is realized.

The heterodyne laser source splits the amplitude through a beam splitter (BS), and the reflected beam carries a beat frequency signal of 80 MHz. It can be detected by an avalanche photodiode (APD), as a reference signal, after the analyzer is used. This reference signal can be transformed into the differential signals, which is mentioned in Section 2.2.4.

The transmitted beam enters the interior of the positioning stage. A BS is inside the stage, to split the amplitude of the laser beam into the transmitted and reflected beams. The reflected beam from the BS transmits to a half-phase retarder, which has an orientation of 45 degrees. This retarder can rotate the polarizing direction of the two laser beams by 90 degrees. Then, those rotated laser beams transmit after the PBS to the two APDs as the reference beam. The transmitted beam enters the measurement area through the PBS, which is inside the stage. The measuring mirrors reflect the split beams to the PBS. There are two quarter-phase retarders, with an orientation of 45 degrees, in the measurement arms. These retarders rotate the polarizing direction of the laser beams by 90 degrees. In this way, these laser beams can reach the APD as the measurement beams.

**Figure 11:**
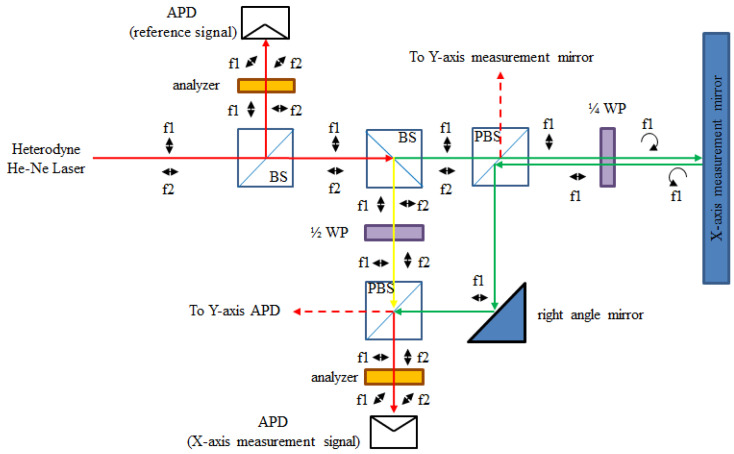
Optical path of X-axis.

The two pairs of reference beams and measuring beams are overlapping at two APDs, as the measurement signals. By the phase-detection modules, these measurement signals and reference signals transform into the displacement information of the X and Y axes. The optical path of the X-axis is discussed in Figure 11. The beam directions, frequencies, and polarizing directions of each route are revealed in those figures. The beam path of the system can be divided into the reference arm (yellow path, d_1_) and the measuring arm (green path, d_2_). The intensity equation of the reference signal (I_r_) is shown in Equation (4). The interference signal of the X-axis (I_x_) is shown in Equations (5) and (6), where λ_1_ and λ_2_ are the wavelengths corresponding to the laser frequencies f_1_ and f_2_, respectively, and ϕ_x_ is the phase difference between the paths d_1_ and d_2_.

(4) $$I_{r}\left(t\right)=A_{r}\cdot \cos\left[2\pi t\left(f_{1}-f_{2}\right)\right]$$

(5) $$I_{x}\left(t\right)=A_{x}\cdot \cos\left[2\pi t\left(f_{1}-f_{2}\right)+\emptyset_{x}\right]$$

(6) $$\emptyset_{x}=2\pi \left(\frac{d_{1}}{\lambda_{2}}-\frac{d_{2}}{\lambda_{1}}\right)$$

**Figure 12:**
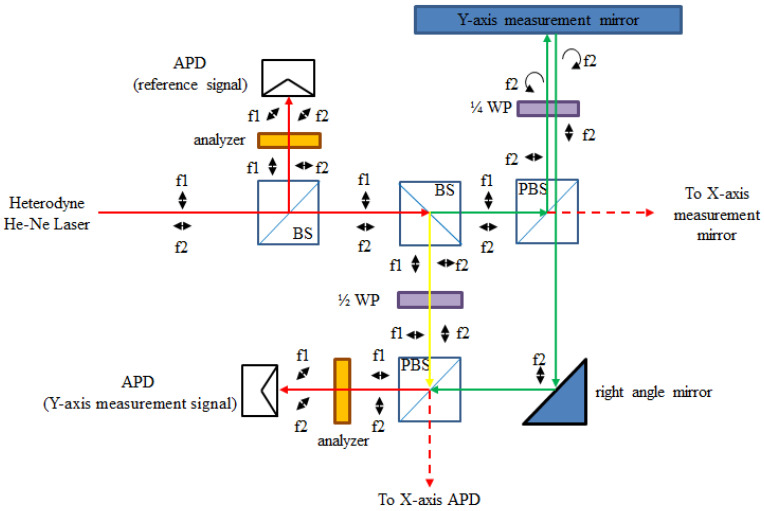
Optical path of Y-axis.

Figure 12 shows the optical path of the Y-axis. The beam path in the system is also divided into the reference arm (yellow path, d_1_) and the measuring arm (green path, d_3_). The reference signal (I_r_) is the same as the *X*-axis. The intensity equation of the Y-axis (I_y_) is shown in Equations (7) and (8). Through this optical, mechanical design, the displacements of the X and Y axes can be realized by analyzing the phase changing of ϕ_x_ and ϕ_y_. Assuming that the reference arm path d_1_ is fixed, the displacements of the measurement arm of d_2_ and d_3_ can be obtained. It can be understood, from Equations (6) and (8), that the frequencies of the X-axis and Y-axis are opposite to the reference beam and the measuring beam. Therefore, during the signal processing, the displacements of the X and Y axes will be linked to the signal. In this way, a real-time encoding system of the X–Y positioning stage can be realized. It is worth mentioning that, due to the frequency difference between the lasers, there is a slight difference between the resolutions of the X- and Y-axis. This special design is the most important to the originality of this research. These two laser frequencies can be used as reference and measurement arms for each other. Also, the reference signal can be shared to the signal processing of the X-axis and Y-axis. In this way, the optical structure can be made simpler.

(7) $$I_{y}\left(t\right)=A_{y}\cdot \cos\left[2\pi t\left(f_{1}-f_{2}\right)+\emptyset_{y}\right]$$

(8) $$\emptyset_{y}=2\pi \left(\frac{d_{1}}{\lambda_{1}}-\frac{d_{3}}{\lambda_{2}}\right)$$

### 3.3. Signal Processing Module

**Figure 13:**
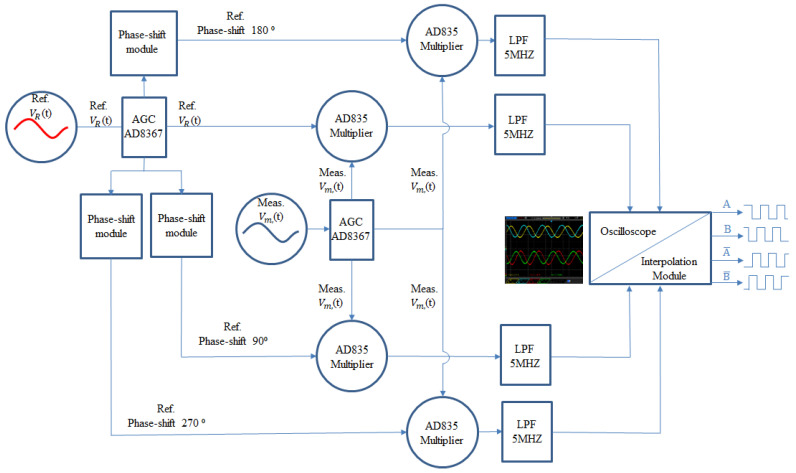
Design of the heterodyne signal processing.

**Figure 14:**
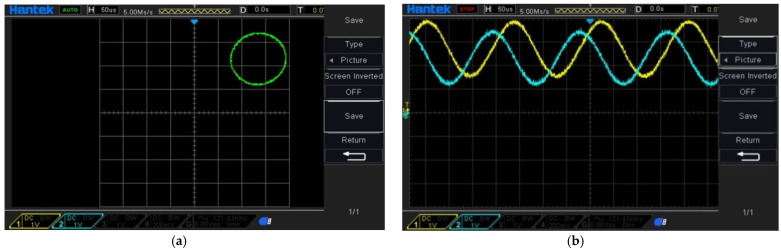
Testing of the interference signal (**a**) Lissajous figure, (**b**) time-domain signal.

Figure 13 shows the design of the heterodyne signal processing module of this study. First, the reference and measurement signals are automatically gained by the AD8367 AGC module, which were developed by Analog Device Company, and have the bandwidth of 500 MHz. Then, the reference signals are delayed by the phase-shift modules for the differential reference signals. Second, the gained measurement signal and the differential reference signal are transmitted into four AD835 multipliers, respectively. These multipliers, with a bandwidth of 250 MHz, were developed by Analog Device Company. Then, deal these multiplied signals with the low-pass filters, to obtain the phase-difference signals. In the end, the phase-difference signal is transmitted to the oscilloscope and interpolation module, for observing and encoding the displacement signals. The Lissajous figure and the time-domain signal of the oscilloscope are shown in Figure 14.

## 4. Experimental Results and Analysis

**Figure 15:**
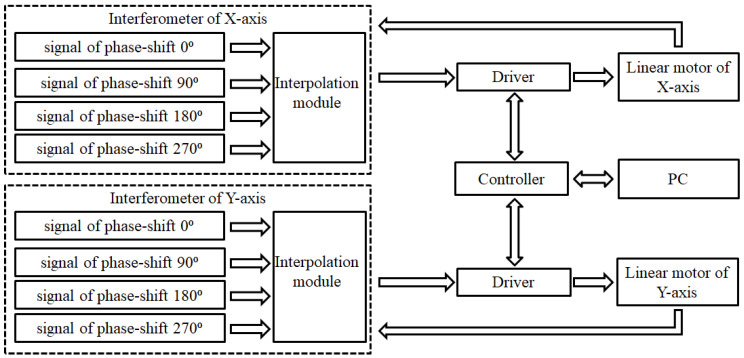
Sketch of the feedback control system.

The experiment in this article uses the above-mentioned optical structure, with an internal zero-point design. The signal processing subdivides the laser encoder signal by 20 times, by the subdivision module. In this way, the resolution of the proposed laser encoder is about 15.8 nm. This encoding signal can be the feedback resource to the driver and controller. Therefore, the closed-loop control of the positioning stage in high accuracy can be realized with the proposed encoder system. The sketch of the feedback control system is shown in Figure 15.

### 4.1. Experiment Result of X-Axis

**Figure 16:**
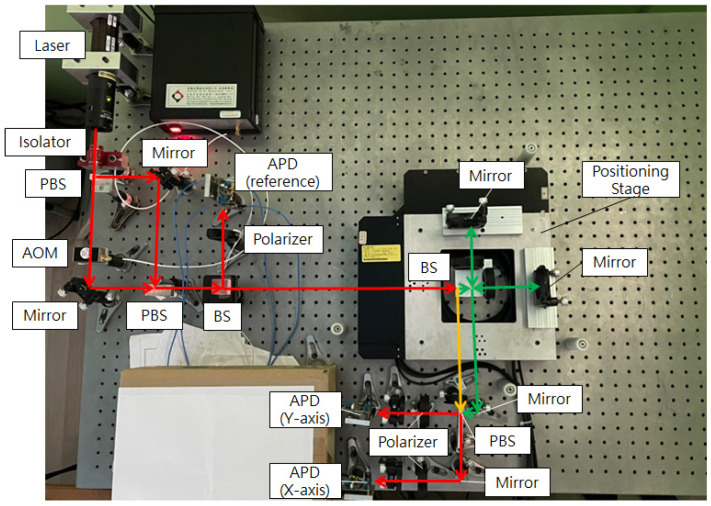
Experimental structure.

**Figure 17:**
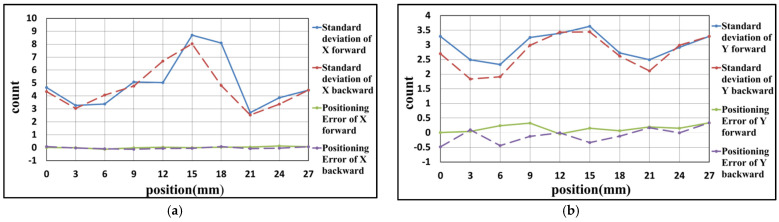
Result of the first positioning experiment of X-axis (**a**) X-axis (**b**) Y-axis.

**Figure 18:**
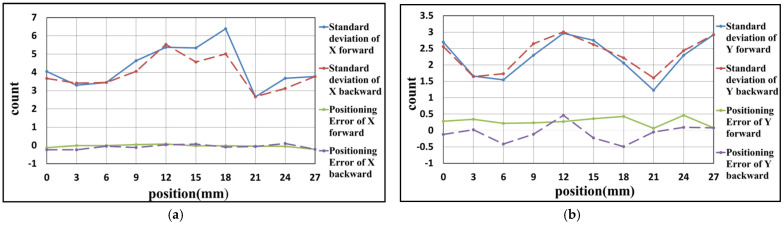
Result of the second positioning experiment of X-axis (**a**) X-axis (**b**) Y-axis.

**Figure 19:**
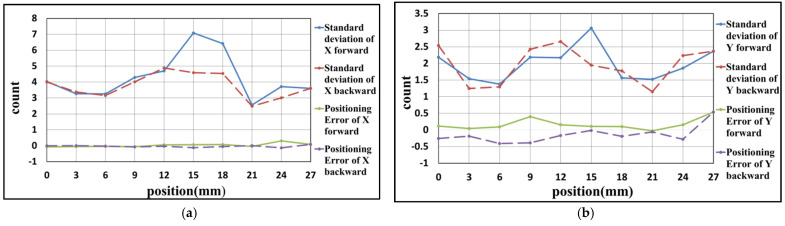
Result of the third positioning experiment of X-axis (**a**) X-axis (**b**) Y-axis.

Due to the limitation of the experimental space, some slight change in the optical design was made to the experimental structure, as shown in Figure 16. These changes are convenient for the setup of the experiment and do not affect the previous theoretical analysis. The results of the linear positioning experiments for the X-axis are shown as follows. There are three rounds of experiments, with the positioning range from 0 to 27 mm, and the positioning interval is 3 mm. When the linear positioning is being processed, the closed-loop control of the Y-axis is executed simultaneously. After three rounds of experiments, the results are carried out, as shown in Figure 17, Figure 18 and Figure 19 and Table 2, Table 3 and Table 4. The maximum positioning error and the maximum standard deviation of the X-axis are about 0.4 counts (6.32 nm) and 8 counts (126.4 nm), respectively. The maximum positioning error and the maximum standard deviation of the Y-axis are about 0.6 counts (9.48 nm) and 4 counts (63.2 nm), respectively.

### 4.2. Experiment Result of Y-Axis

**Figure 20:**
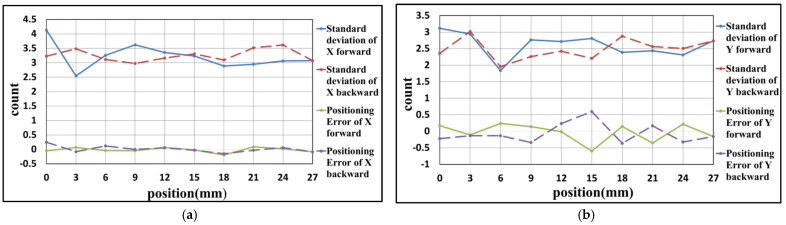
Result of the first positioning experiment of Y-axis (**a**) X-axis (**b**) Y-axis.

**Figure 21:**
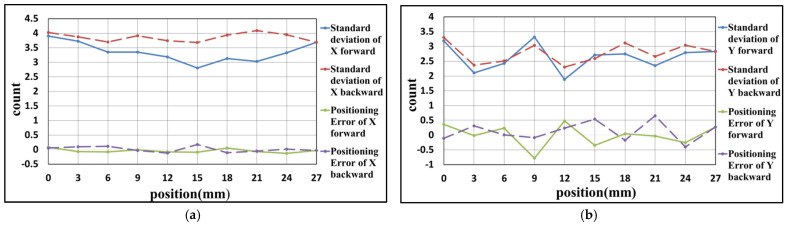
Result of the second positioning experiment of Y-axis (**a**) X-axis (**b**) Y-axis.

**Figure 22:**
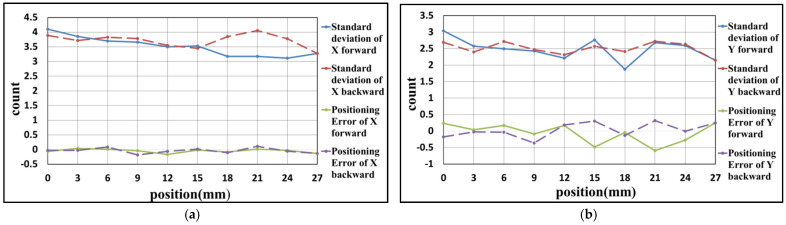
Result of the third positioning experiment of Y-axis (**a**) X-axis (**b**) Y-axis.

In this experiment, the positioning axis is changed to the Y-axis of the stage. The positioning range and interval are the same as the experiment in Section 4.1. The results are carried out, as shown in Figure 20, Figure 21 and Figure 22 and Table 5, Table 6 and Table 7. These results show that the maximum positioning error and the maximum standard deviation of the X-axis are about 0.3 counts (4.74 nm) and 4 counts (63.2 nm), respectively. The maximum positioning error and the maximum standard deviation of the Y-axis are about 0.8 counts (12.64 nm) and 4 counts (63.2 nm), respectively.

### 4.3. Analysis of the Experimental Results

By the experimental results, the positioning errors are within one count (15.8 nm) during the whole experiment. This means that the interaction error of multiple axes could be compensated by the proposed encoder system. The standard deviations of the X-axis are a little bit higher than those in the Y-axis. The reason for this is that the alignment of the Y-axis is better than the X-axis. Therefore, an alignment tool and procedure should be investigated in future work; it will be the key to the next level for this encoder system. The positioning range of the target stage is limited by the length of the reflection mirrors. Once the mirror is enlarged, the positioning range could reach more than 100 mm, which we have tested on a single axis before. The precision of the target stage is about 0.5 μm, which is evaluated by the standard deviation with a coverage factor of three. The precision level of this target stage could meet the requirement of the positioning task at the sub-micrometer scale. In the nanometer scale, some further works should be conducted, such as the alignment issue, the control parameters, and the mechanical design of the stage.

## 5. Conclusions

In this research, a heterodyne laser encoder system and its application has been proposed. According to the experimental results, the two-dimensional laser encoder system has expectant potential in the precision machinery and semiconductor industries. The contributions of this research are shown as follows:

1. Referring to the references of the Abbe error-free design, this research has proposed a geometric error-reducing design for the heterodyne laser encoder in the two-dimensional stage. This embedded optical, mechanical design can improve the accuracy of the positioning stage. More importantly, the proposed method can be implemented in the commercial controllers, without any complicated compensation algorithm;
2. By using the commercial integrated circuit (IC), the signal processing system of the heterodyne interferometer has been realized. This system, including the AGC module, phase-shifting module, and phase-detection module, can generate the differential encoding signal for the feedback control system. In this way, the proposed encoding system is more competitive in industrial applications;
3. A series of positioning experiments in an ordinary environment has been carried out. In the experiment range of 27 mm × 27 mm, the system resolution is about 15.8 nm. The experimental result shows that the maximum values of the positioning error and standard deviations are 6.32 nm and 126.4 nm for the X-axis, respectively, and the maximum values of the positioning error and standard deviations are 12.64 nm and 63.2 nm in the Y-axis, respectively. According to the experimental results, this proposed encoder system can meet the application requirements of the optoelectronic and semiconductor industries.

## Figures and Tables

**Table 1:** Comparison of the precision positioning technologies.

Feature Technology	Optical Structure	Laser Source	Geometric Error Reducing Method	Assembly/Alignment Requirement	System Cost	Degree of Freedom (Dof) of Positioning System	Signaling Design	Application Field
Proposed heterodyne laser encoder system	Embedded Michelson-like interferometer	Heterodyne laser	Internal zero-point method	Laser alignment and optical elements assembly	Medium	2 DOF	Shared reference signal	2D positioning
Previous system based on internal zero-point method [24]	Embedded Fabry–Perot interferometer	Homodyne laser	Internal zero-point method	Laser alignment and optical elements assembly	Medium	2 DOF	Individual reference signal	2D positioning
Previous system based on external zero-point method [21,22,23]	Multiple Michelson interferometer sensors	Homodyne/heterodyne laser	External zero-point method	Multiple sensor heads assembly and zero-point alignment	High	6 DOF	Individual reference signal	3D positioning
Commercial encoder system of optical grating interferometer	Multiple Optical grating interferometer sensors	Laser diode	Being compensated by error analysis algorithm	Based on the bearing surface	Low	2 or 3 DOF	Individual reference signal	2D or 3D positioning

**Table 2:** Result of the first positioning experiment of X-axis.

Position (mm)	Positioning Error of X-Axis	Standard Deviation of X-Axis	Positioning Error of Y-Axis	Standard Deviation of Y-Axis
0	0.02	4.66	0.01	3.29
3	0.01	3.27	0.04	2.49
6	−0.12	3.38	0.24	2.33
9	−0.01	5.08	0.33	3.26
12	0.03	5.03	−0.04	3.39
15	0.00	8.70	0.15	3.63
18	0.04	8.09	0.07	2.73
21	0.06	2.71	0.20	2.49
24	0.13	3.86	0.15	2.92
27	0.07	4.45	0.34	3.29
24	−0.04	3.37	−0.01	2.99
21	−0.07	2.53	0.17	2.11
18	0.09	4.81	−0.12	2.62
15	−0.05	8.03	−0.34	3.44
12	−0.05	6.70	0.00	3.43
9	−0.12	4.76	−0.13	2.99
6	−0.09	4.07	−0.44	1.91
3	−0.02	3.04	0.10	1.84
0	0.08	4.34	−0.48	2.70

**Table 3:** Result of the second positioning experiment of X-axis.

Position (mm)	Positioning Error of X-Axis	Standard Deviation of X-Axis	Positioning Error of Y-Axis	Standard Deviation of Y-Axis
0	−0.12	4.05	0.28	2.69
3	0.00	3.30	0.34	1.66
6	0.00	3.45	0.22	1.55
9	0.05	4.64	0.24	2.29
12	0.08	5.37	0.28	2.97
15	−0.02	5.34	0.36	2.75
18	−0.02	6.39	0.43	2.06
21	−0.04	2.66	0.07	1.23
24	−0.05	3.68	0.46	2.30
27	−0.21	3.78	0.08	2.92
24	0.11	3.12	0.10	2.44
21	−0.06	2.68	−0.04	1.60
18	−0.09	5.02	−0.49	2.22
15	0.07	4.57	−0.23	2.62
12	0.04	5.53	0.47	3.01
9	−0.11	4.06	−0.12	2.65
6	−0.04	3.44	−0.41	1.73
3	−0.23	3.41	0.02	1.64
0	−0.23	3.67	−0.12	2.56

**Table 4:** Result of the third positioning experiment of X-axis.

Position (mm)	Positioning Error of X-Axis	Standard Deviation of X-Axis	Positioning Error of Y-Axis	Standard Deviation of Y-Axis
0	−0.07	4.04	0.12	2.19
3	−0.05	3.27	0.04	1.54
6	−0.04	3.26	0.09	1.38
9	−0.06	4.30	0.40	2.19
12	0.06	4.70	0.16	2.17
15	0.07	7.08	0.11	3.06
18	0.08	6.42	0.10	1.56
21	−0.04	2.57	−0.02	1.52
24	0.31	3.71	0.16	1.86
27	0.09	3.60	0.54	2.37
24	−0.13	3.01	−0.28	2.24
21	0.01	2.49	−0.06	1.15
18	−0.05	4.54	−0.19	1.78
15	−0.12	4.59	−0.02	1.95
12	−0.04	4.89	−0.17	2.66
9	−0.07	4.02	−0.39	2.43
6	−0.02	3.16	−0.41	1.30
3	0.00	3.38	−0.19	1.25
0	0.00	4.01	−0.26	2.54

**Table 5:** Result of the first positioning experiment of Y-axis.

Position (mm)	Positioning Error of X-Axis	Standard Deviation of X-Axis	Positioning Error of Y-Axis	Standard Deviation of Y-Axis
0	−0.05	4.13	0.17	3.12
3	0.06	2.55	−0.11	2.94
6	−0.04	3.26	0.24	1.84
9	−0.04	3.62	0.14	2.77
12	0.07	3.36	−0.01	2.72
15	−0.02	3.23	−0.60	2.81
18	−0.19	2.89	0.14	3.12
21	0.09	2.95	−0.35	2.44
24	0.02	3.06	0.22	2.31
27	−0.09	3.08	−0.16	2.74
24	0.06	3.61	−0.33	2.50
21	−0.03	3.52	0.16	2.57
18	−0.15	3.1	−0.36	2.88
15	−0.03	3.31	0.60	2.21
12	0.05	3.16	0.24	2.42
9	−0.12	4.76	−0.13	2.99
6	−0.09	4.07	−0.44	1.91
3	−0.02	3.04	0.10	1.84
0	0.08	4.34	−0.48	2.70

**Table 6:** Result of the second positioning experiment of Y-axis.

Position (mm)	Positioning Error of X-Axis	Standard Deviation of X-Axis	Positioning Error of Y-Axis	Standard Deviation of Y-Axis
0	0.08	3.91	0.36	3.19
3	−0.07	3.73	−0.01	2.11
6	−0.08	3.35	0.24	2.43
9	0.00	3.35	−0.78	3.32
12	−0.08	3.19	0.48	1.88
15	−0.09	2.80	−0.35	2.71
18	0.06	3.13	0.05	2.75
21	−0.07	3.03	−0.03	2.35
24	−0.13	3.33	−0.25	2.79
27	−0.03	3.69	0.28	2.83
24	0.02	3.95	−0.40	3.04
21	−0.05	4.09	0.65	2.66
18	−0.11	3.94	−0.17	3.12
15	0.18	3.68	0.54	2.59
12	−0.12	3.74	0.24	2.30
9	−0.03	3.92	−0.09	3.04
6	0.11	3.70	0.01	2.51
3	0.10	3.88	0.31	2.37
0	0.06	4.03	−0.11	3.30

**Table 7:** Result of the third positioning experiment of Y-axis.

Position (mm)	Positioning Error of X-Axis	Standard Deviation of X-Axis	Positioning Error of Y-Axis	Standard Deviation of Y-Axis
0	−0.06	4.10	0.23	3.04
3	0.04	3.85	0.04	2.58
6	0.01	3.70	0.17	2.50
9	−0.04	3.66	−0.09	2.43
12	−0.17	3.50	0.17	2.21
15	−0.02	3.53	−0.48	2.76
18	−0.08	3.17	−0.05	1.87
21	0.02	3.18	−0.59	2.68
24	−0.03	3.11	−0.27	2.59
27	−0.13	3.28	0.25	2.15
24	−0.06	3.77	−0.01	2.63
21	0.11	4.05	0.32	2.72
18	−0.10	3.85	−0.13	2.41
15	0.02	3.45	0.30	2.57
12	−0.06	3.55	0.18	2.31
9	−0.18	3.78	−0.36	2.47
6	0.09	3.83	−0.04	2.71
3	−0.03	3.72	−0.03	2.40
0	−0.03	3.89	−0.18	2.69

## Data Availability

Not applicable.

## References

[1] Wu M.-H., Fang Y.-H. (2016). Picking-up and Placing Process for Electronic Devices and Electronic Module. U.S. Patent.

[2] Mizuno T., Tomoda K., Oohata T. (2010). Method of Transferring Device. U.S. Patent.

[3] Golda D., Higginson J.A., Bibl A., Parks P.A., Bathurst S.P. (2016). Mass Transfer Tool Manipulator Assembly. U.S. Patent.

[4] Li Y.-C., Lai Y.-H., Lin T.-Y. (2017). Method for Transferring Light-Emitting Elements onto a Package Substrate. U.S. Patent.

[5] Maiman T.H. (1960). Stimulated Optical Radiation in Ruby. Nature.

[6] BIPM (1983). 17th General Conference on Weights and Measures, Resolution 1.

[7] Suh Y.S. (2019). Laser Sensors for Displacement, Distance and Position. Sensors.

[8] Hori Y., Gonda S., Bitou Y., Watanabe A., Nakamura K. (2018). Periodic Error Evaluation System for Linear Encoders Using a Homodyne Laser Interferometer with 10 Picometer Uncertainty. Precis. Eng..

[9] Yan L., Chen1 B., Chen Z., Xie J., Zhang E., Zhang S. (2017). Phase-Modulated Dual-Homodyne Interferometer without Periodic Monlinearity. Meas. Sci. Technol..

[10] Fu H., Ji R., Hu P., Wang Y., Wu G., Tan J. (2018). Measurement Method for Nonlinearity in Heterodyne Laser Interferometers Based on Double-Channel Quadrature Demodulation. Sensors.

[11] Zhang E., Chen B., Zheng H., Yan L., Teng X. (2018). Laser Heterodyne Interferometer with Rotational Error Compensation for Precision Displacement Measurement. Opt. Express.

[12] Michels W.C., Curtis N.L. (1941). A Pentode Lock-In Amplifier of High Frequency Selectivity. Rev. Sci. Instrum..

[13] Stutt C.A. (1949). Low-Frequency Spectrum of Lock-in Amplifiers. MIT Tech. Rep. (MIT).

[14] De Graaf G., Wolffenbuttel R.F. Lock-in Amplifier Techniques for Low-frequency Modulated Sensor Applications. Proceedings of the 2012 IEEE International Instrumentation and Measurement Technology Conference Proceedings.

[15] Ibrahim S.M.M., Saedon J., Radzi A., Omar R. (2019). Improvement of Positional Accuracy of Developed Dicing Machine. Int. J. Mech. Eng. Robot. Res..

[16] Maj P., Miko E. Linear and Angle Displacement Measurement of Vertical Milling Center Using a Multi-Axis Calibrator. Proceedings of the 24th International Conference Engineering Mechanics 2018.

[17] Jia Z., Ma J., Song D., Wang F., Liu W. (2018). A Review of Contouring-error Reduction Method in Multi-axis CNC Machining. Int. J. Mach. Tools Manuf..

[18] Zhang S., Lu S., He R., Bao Z. (2021). Stereo Visual Odometry Pose Correction through Unsupervised Deep Learning. Sensors.

[19] Andriyanov N., Vasiliev K. (2020). Using Local Objects to Improve Estimation of Mobile Object Coordinates and Smoothing Trajectory of Movement by Autoregression with Multiple Roots. IntelliSys 2019: Intelligent Systems and Applications.

[20] Bryan J.B. (1979). The Abbe Principle revisited: An Updated Interpretation. Precis. Eng..

[21] Ruijl T.A.M., van Eijk J. A Novel Ultra Precision CMM Based on Fundamental Design Principles. Proceedings of the ASPE Topical Meeting on Coordinate Measuring Machines.

[22] Jeager G. (2010). Three-Dimensional Nanopositioning and Nanomeasuring Machine with a Resolution of 0.1 nm. Optoelectron. Instrum. Data Process..

[23] Huang Q., Wu K., Wang C., Li R.-J., Fan K.-C., Fei Y. (2016). Development of an Abbe Error Free Micro Coordinate Measuring Machine. Appl. Sci..

[24] Chang C.P., Shih Y.-C., Chang S.-C., Wang Y.-C. (2019). Laser Encoder System for X-Y Positioning Stage. Mechatronics.

[25] Jeager G. (2010). Limitations of Precision Length Measurements Based on Interferometers. J. Meas..

[26] Topcu S., Chassagne L., Haddad D., Alayli Y. (2003). Heterodyne Interferometric Technique for Displacement Control at the Nanometric Scale. Rev. Sci. Instrum..

[27] Huang Q., Liu X., Sun L. Homodyne Laser Interferometric Displacement Measuring System with Nanometer Accuracy. Proceedings of the Ninth International Conference on Electronic Measurement & Instruments.

[28] Rabijns D., Moer W.V., Vandersteen G. (2003). A Full Grown Differential Signal Source. ARFTG Microw. Meas..

[29] Chen Z., Katopis G. A Comparison of Performance Potentials of Single Ended vs. Differential Signaling. Proceedings of the Electrical Performance of Electronic Packaging-2004.

